# Assessment of Clients' Perceptions and Satisfaction With Eyebrow Mesotherapy Treatment

**DOI:** 10.1111/jocd.16699

**Published:** 2024-11-27

**Authors:** Krenar Dobroshi, Marija Glavash Dodov, Maja Simonovska Crcarevska, Renata Slaveska Raichki, Selvete Shuleta‐Qehaja, Blerim Krasniqi

**Affiliations:** ^1^ Alma Mater Europaea College of Medical Sciences “Rezonanca” Prishtina Albania; ^2^ The Saints Cyril and Methodius University Faculty of Pharmacy Skopje North Macedonia

## Abstract

**Background:**

Eyebrows significantly influence facial aesthetics and are often linked to attractiveness and personality. Eyebrow loss and alopecia impact physical and psychological well‐being. Several treatments, including mesotherapy (MT), aim to enhance eyebrow density and quality. Despite the availability of MT for hair growth, its application in eyebrow revitalization is less explored.

**Aims:**

This study evaluates clients' perceptions and satisfaction with eyebrow MT (EB MT) using AQ skin solutions' growth factor‐based MT cocktail serum.

**Patients/Methods:**

Thirty‐two healthy women (ages 21–55) with thin or weakened eyebrows underwent three EB MT sessions spaced 2–4 weeks apart. Exclusion criteria included chronic diseases, allergies, and conditions like pregnancy. Each session lasted about 40 min, and “point by point” and “nappage” techniques were employed. Satisfaction and pain levels were assessed through a Numeric Rating Scale for Satisfaction (NRSS) and Pain scale, while photographic analysis and the Global Aesthetic Improvement Scale (GAIS) evaluated effectiveness.

**Results:**

The average GAIS score was 8.28, while the NRSS satisfaction score was 8.06. Pain levels were moderately low, averaging 4.0. A high majority (90.6%) were satisfied and willing to continue treatment, with 100% recommending EB MT to others. Notable improvements in overall appearance were reported by participants.

**Conclusions:**

EB MT demonstrated high satisfaction and low pain levels, with significant improvements in eyebrow density and appearance. This treatment is a practical option for eyebrow enhancement with a strong safety profile, motivating further clinical application.

## 1 Introduction

As acknowledged, the main biological function of the EBs is to shield the eyes from light/sun exposure and to protect them from perspiration and environmental factors. On average, the EBs have a length of 4.5–5.5 cm and a height of 0.5–1 cm, they are short in length, small in diameter, and slow growing. EB is composed of 300 to 600 hairs, and it is divided into three parts: the head, the central body, and the lateral tail. The structure, morphogenesis, and function of the EB hair follicle have the same basic characteristics as hair follicles elsewhere on the body; however, they are distinguished by their shorter anagen phase [1, 2].

Moreover, EBs have their own share in the expression of feelings and help people in the communication processes. Furthermore, over the years, the aesthetic functions of eyebrows have also been paid considerable attention. Actually, we live in a society where the perception of our image predominates. On the other hand, the trend of long and full eyebrows is often synonymous with healthy and beautiful skin, overall appearance, and health condition of the body.

However, it is known that temporary or permanent hair loss often leads to serious psychological problems [3]. For instance, Alopecia areata totalis could be one example of a condition where all the hair falls from the skin [4]. Thus, EB regrowth treatment is not always a simple matter, and it has to go through different stages designed and approached by a medical practitioner or trichologist.

Nowadays, the beauty industry and cosmetic clinics offer different techniques for EB treatment. Numerous procedures have been introduced by nonmedical and medical practitioners. For example, these range from pencil correction (which could be erased in contact with water or harsh rubbing), tattooing (which could be often regarded as fake eyebrows), transplantation (an invasive surgical technique) to mesotherapy (MT).

By definition, MT refers to a nonsurgical, minimally invasive injection technique for substance delivery into a targeted intradermal and subcutaneous tissue [5]. The combination of substances injected depends on the mixture choice made by the medical practitioner and the intended use [6]. In practice, the MT is a widely implemented procedure to treat areas of unwanted fat accumulation or cellulite [7]; furthermore, in the field of body contouring [8], chronic pain management [9], bone and joint disorders [10], and among the list is psoriasis [11]. More recently, MT has been used for wrinkle removal, facial rejuvenation, revitalization, as well as hair‐stimulating regrowth of fallen hair follicles [12].

Although there have been many studies in the domain of MT until now, a specific address of MT use in EB treatment is still infrequent [13]. Therefore, at this stage of our study, we accept the challenge of assessing the clients' perception and satisfaction with developed EB MT treatment. Another supportive reason for our study is the increasing demand for EB treatment.

## 2 Materials and Methods

### 2.1 Client Selection

The study includes 32 healthy women volunteers/clients (aged between 21 and 55 years old). Distribution by age comprises Group I (12 clients) aged between 21 and 31 years; Group II (7 clients) aged between 32 and 42 years; Group III (13 clients) aged between 43 and 53 years; with thin, weakened, fallen EBs or reduced volumes of EBs. Actually, the clients experiencing minimal (< 25%), moderate (25%–50%), or severe eyebrow hair loss (> 50%) are our target groups. The clients were recruited from the Aesthetic and Dermatology Clinic “Estethica” in Prishtina. All the EB MT sessions were performed in this Clinic from October 2018 to September 2023. The study was paused during a pandemic period. All clients in this study gave informed written consent for the EB MT procedures and filled in an extensive medical questionnaire under the supervision of a physician. Clients with diagnosed diabetes, cancer, blood diseases, septicemia, and AIDS were excluded including anticoagulated clients and those with any allergy or hypersensitivity to any ingredients present in the MT cocktail used in EB MT.

### 2.2 EB MT Protocol

Each client has had three sessions of EBs MT every 2–4 weeks apart, with or without topical anesthetic cream (4% lidocaine) depending on their pain tolerance. The application time for each EB MT session was about 40 min, 10 min for each eyebrow treatment, 5 min for disinfection, and another 15 min for absorption of the cocktail.

The EB MT cocktail/serum used is AQ advance hair complex, AQ Skin Solutions Inc. (Irvine, California), which contains substances that stimulate hair regrowth [14, 15].

### 2.3 MT Techniques and Application Device

“Point by point” and “nappage” techniques were applied. Both techniques were performed by using the MT professional device (mesopen, MyM) with disposable tip sterile microneedles (replacement cartage, 12 microneedles size 2.5 mm) which penetrate the skin from 2.0 to 3.0 mm [16].

### 2.4 Eyebrow Serum/Cocktail Selection Criteria

Before we had chosen AQ advance hair complex, AQ Skin Solutions Inc. (Irvine, California), we did extensive research regarding the best outcomes and most studied product that would potentially enhance and strengthen the eyebrows. We wanted to have a product that contains growth factors without any growth hormones. AQ Skin Solutions products are the only ones that met our criteria. These standards are unique to only AQ products. This has also been confirmed when we ran a ChatGPT search asking it about the most efficient and effective growth factors product in the market. The answer was AQ Skin Solutions products.

Criteria that made us choose AQ Skin Solutions growth factors:

1. The growth factors produced by AQ Skin Solutions Inc. (Irvine, California) have been used and referenced in many publications affirming their effectiveness in hair growth and regeneration [14, 15];
2. The AQ growth factors are manufactured in a GMP and FDA‐registered facility ensuring high‐quality production standards;
3. These growth factors are produced under rigorous pharmaceutical standards, enhancing their reliability and safety;
4. AQ Skin Solutions products have a proven safety record in clinical use, which reassured us of their appropriateness for our study.

We felt safe and comfortable to pick a product which has been studied before and published on.

### 2.5 Evaluation Protocol

Each client filled up a Client Satisfaction Questionnaire (Appendix A), developed for the purposes of this study, after every MT session, after the 4 weeks of the last session, and at the 6‐month follow‐up, where they scored the overall appearance of the treated EB area, along with its perception and satisfaction on a 10‐point scale. Clients also provided feedback on their pain experience during the EB MT procedure; noticeable pain (scored from 1 to 3), moderately low (scored from 4 to 6), and strong pain (scored from 7 to 10). Any adverse events were closely monitored by the physician from the Aesthetic and Dermatology Clinic “Estethica” in Prishtina. Moreover, clients were also asked to score their overall satisfaction and if they would have the MT procedure repeated in the future.

Changes in eyebrow improvement were followed using standardized digital photographs (a total of 6 pictures). Three initial photographs were taken before the first session, from different angles. One photograph is taken in total perspective, and two more on the right and left sides, respectively. At least 4 weeks after the last session, three more photographs are taken from the same perspective and compared with the initial ones. The photos were assessed independently by a third party.

## 3 Results and Discussion

An extensive medical questionnaire developed for the purpose of the study provides information about the client's health status. Obtained data were assessed for inclusion and exclusion criteria. It is also considered that MT is not recommended during pregnancy and breastfeeding [17].

Evidence reporting of possible intolerances or side effects after each session revealed that no side effect was observed after each MT session beyond a little redness around the eyebrows, which disappears in 2–4 h, and a mild level of discomfort. It can be summarized that applied EB MT has an adequate safety profile for the intended use and it is well tolerated.

Generally, there are three main MT techniques: “Papule,” “Nappage,” and “Point by Point”. “Papule” technique is the most superficial and it was not of interest. We have employed both of the techniques “Point by point” and “Nappage.” Even though both of these techniques have shown improvements in the eyebrow MT treatment, based on our previous experiences, we believe that “Point by Point” technique has had more impact. This technique reaches more depth, up to the deep intradermal layer, and by opening the channels through MT needles, the serum was able to better penetrate the hair follicle.

Four weeks after the last session, that is, on the day of the after picture, the clients filled out a questionnaire regarding the overall efficacy of the MT. In total seven straightforward questions were asked.

To measure subjective consumer satisfaction, another rating of NRSS (Numeric Rating Scale for Satisfaction) was used. A rating scale of 0–10 was used, with 0 representing the lowest level of satisfaction and 10 representing the utmost satisfaction. The participants' perceptions are presented in the following figures (1–10).

The effectiveness of the treatment was evaluated through a physician's assessment of before and after treatment photos, applying GAIS (Global aesthetic improvement scale) ranging from 0 to 10. A score of 0 indicated no improvement, while a score of 10 indicated the highest level of improvement.

**FIGURE 1:**
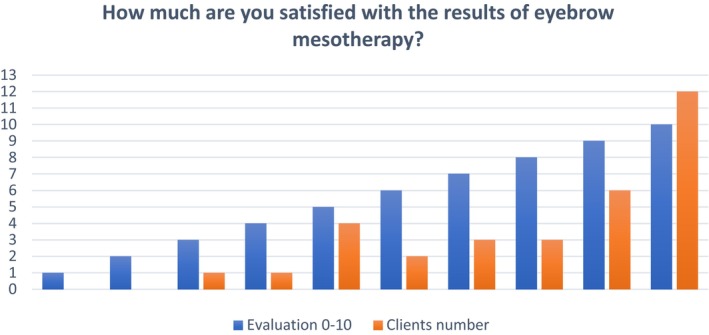
Evaluation of how much clients are satisfied with the results of EB MT.

More than 30% of the clients reported the highest level of satisfaction (score 10) with the results of EB MT Figure 1 Table 1.

**TABLE 1:** Percentage of client's satisfaction with the results of EB MT.

Evaluation 0–10	1	2	3	4	5	6	7	8	9	10
Clients number	0 (0%)	0 (0%)	1 (3.1%)	1 (3.1%)	4 (12.5%)	2 (6.2%)	3 (9.3%)	3 (9.3%)	6 (18.7%)	12 (37.5%)

**FIGURE 2:**
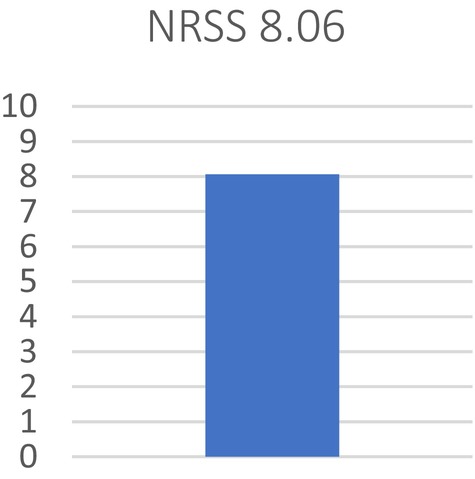
Numeric rating scale for satisfaction assessment.

The average NRSS was 8.06, indicating a very high satisfaction rate (Figure 2).

**FIGURE 3:**
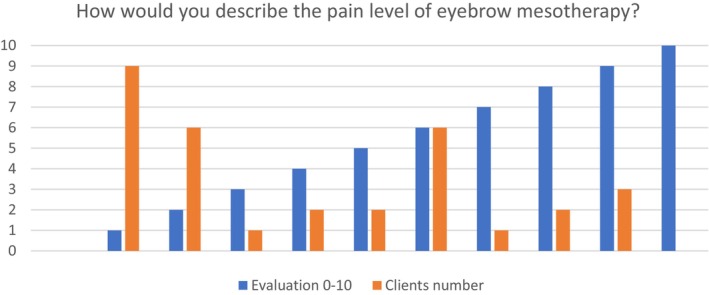
Evaluation of pain level of EB MT.

When clients were asked about their pain level, the obtained average score was 4.0, referring to moderately low pain and high tolerability Figure 3 Table 2.

**TABLE 2:** Evaluation of the pain level 1–10.

Evaluation of the pain level 1–10	1	2	3	4	5	6	7	8	9	10
Clients number	9 (28%)	6 (18.7%)	1 (3.1%)	2 (6.2%)	2 (6.2%)	6 (18.7%)	1 (3.1%)	2 (6.2%)	3 (9.3%)	0

**FIGURE 4:**
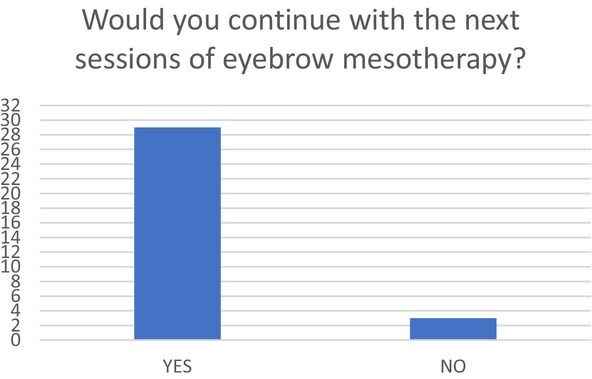
Clients' perception about the continuation of the EB MT session.

Regarding the continuation of the MT sessions, 29 of the clients confirmed that they are willing to continue with further EB MT sessions. Only two of the clients did not rate the first MT session positively. The high approval rate indicates that the majority of the clients (90.6%) are highly satisfied with the first applied session of EB MT protocol Figure 4 Table 3.

**TABLE 3:** Percentage of the clients' perception about the continuation of the EB MT session.

	Yes	No
Clients number	29 (90.06%)	3 (9.37%)

In the next question, all of the 32 clients indicated that they would recommend EB MT to others.

**FIGURE 5:**
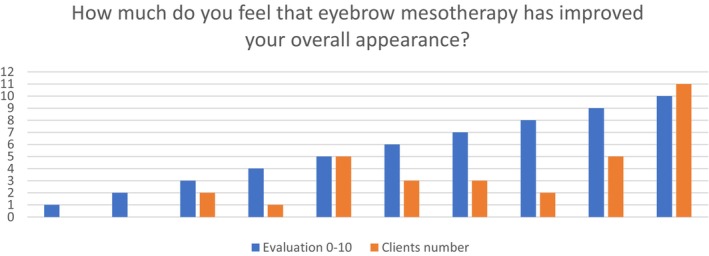
Perception of how much the clients feel that eyebrow mesotherapy has improved their overall appearance.

The last question concerned the client's perception of overall appearance after the completion of EB MT protocol. The majority of the participants scored 5 and above regarding their overall improved appearance. (Figure 2) The average NRSS was 8.06, indicating a very high satisfaction rate Figure 5 Table 4.

**TABLE 4:** Percentage of clients' perception of how EB MT has improved overall appearance.

Overall appearance perception score	1	2	3	4	5	6	7	8	9	10
Clients number	0	0	2 (6.2%)	1 (3.1%)	5 (15.6%)	3 (9.3%)	3 (9.3%)	2 (6.2%)	5 (15.6%)	11 (34.3%)

In the additional suggestions part, only two participants marked their comments as below:

1—I would strongly recommend it to people with eyebrow loss.

2—Perfect treatment.

**FIGURE 6:**
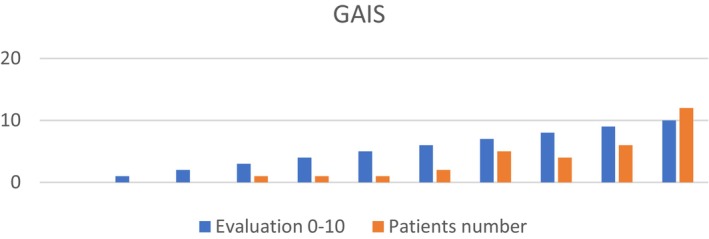
GAIS (Global aesthetic improvement scale).

The effectiveness of the treatment was evaluated through the physician's assessment of before and after EB MT treatment photos (Figure 10), applying GAIS (Global aesthetic improvement scale) ranging from 0 to 10, scoring a motivating average of 8.28 Figure 6 Table 5.

**TABLE 5:** Percentage of GAIS (Global aesthetic improvement scale).

GAIS score	1	2	3	4	5	6	7	8	9	10
Clients number	0	0	1 (3.1%)	1 (3.1%)	1 (3.1%)	2 (6.2%)	5 (15.6%)	4 (12.3%)	6 (18.7%)	12 (37.5%)

**FIGURE 7:**
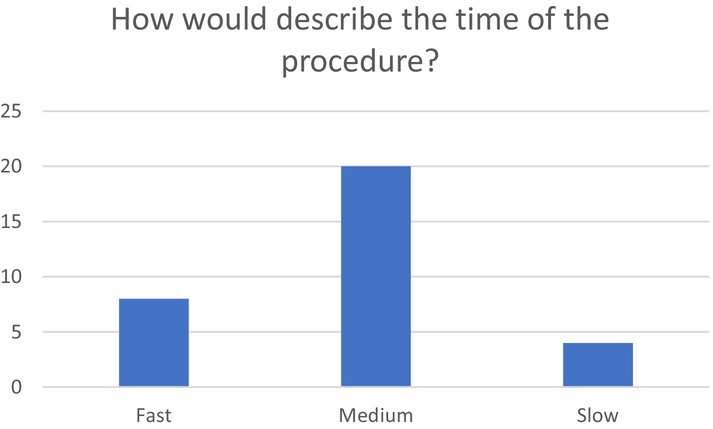
How would you describe the time of the procedure?

Regarding the time of the procedure, most of the clients (20) stated that the time needed for the EB MT was medium in length, second group [8] stated fast, and the smallest group [4] stated slow Figure 7.

Brows grow in a 2‐ to 3‐month cycle. To create an improved biological environment that promotes eyebrow growth, we used mesotherapy (MT) multicomponent serum. In addition, the serum components support hair's natural renewal cycle and speed up the growth process. Thus, trichoscopy and photography analysis after the first session of MT in 32 clients showed thickness and an increase in volume (results not present here). MT can probably give a boost to the already active EB follicles, as well as activate the inactive ones.

**FIGURE 8:**
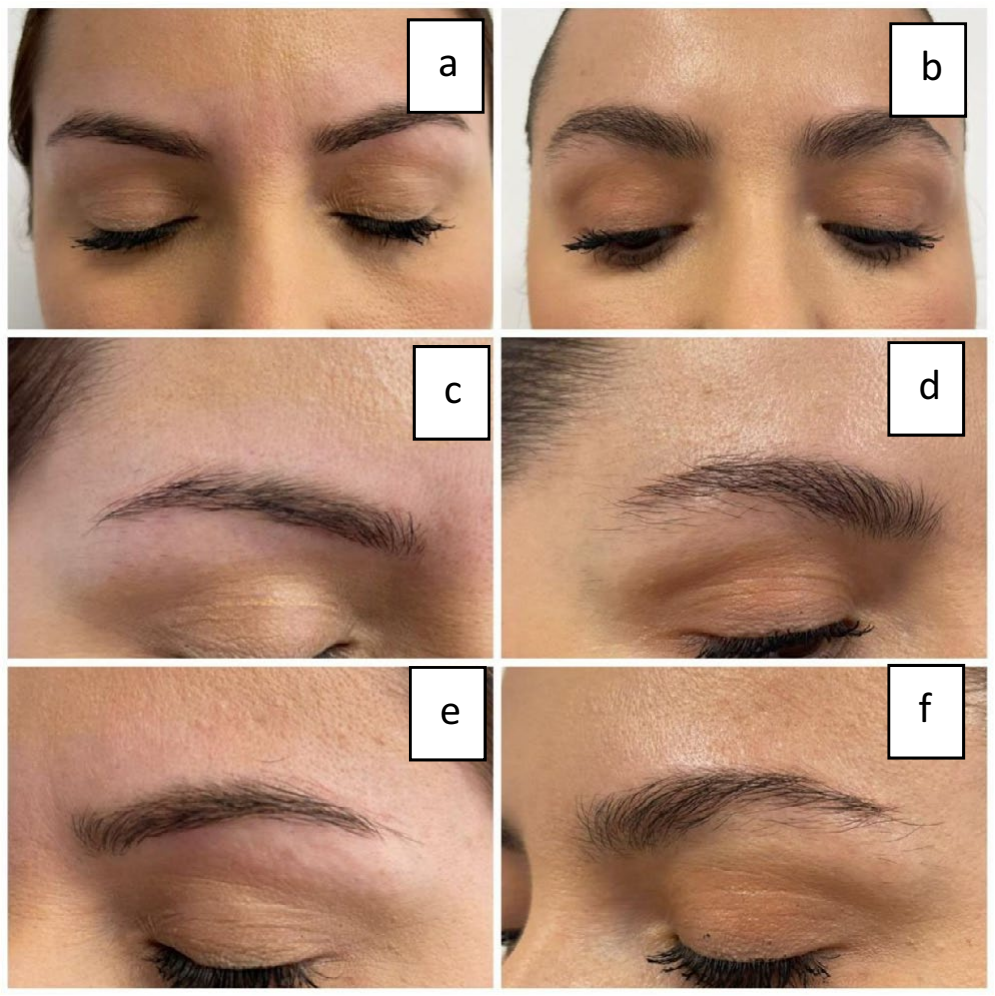
Clinical evaluation of clients with minimal eyebrow loss response to EB MT. Figures a, c, and e represent the before‐first session–baseline from different perspectives: Front and side views; figures b, d, and f represent the after result from different perspectives: Front and side views.

**FIGURE 9:**
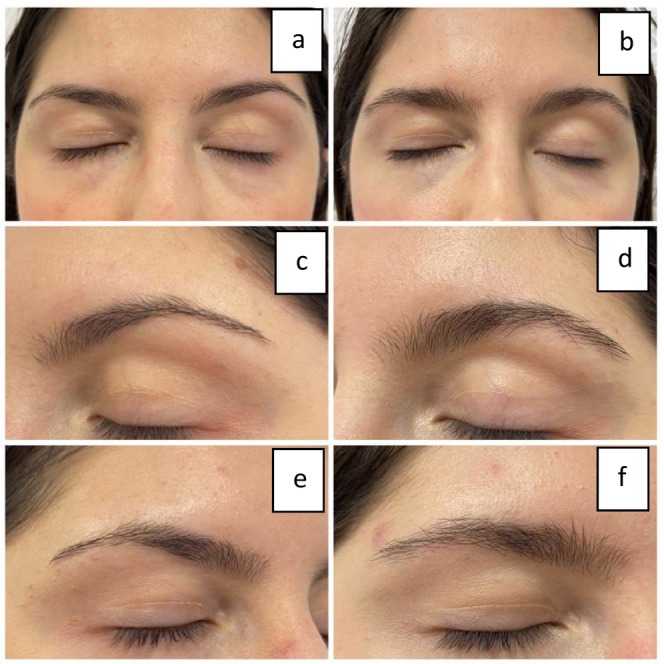
Clinical evaluation of a client with moderate eyebrow loss response to EB MT. Figures a, c, and e represent the before‐first session–baseline from different perspectives: Front and side views; figures b, d, and f represent the after result from different perspectives: Front and side views.

**FIGURE 10:**
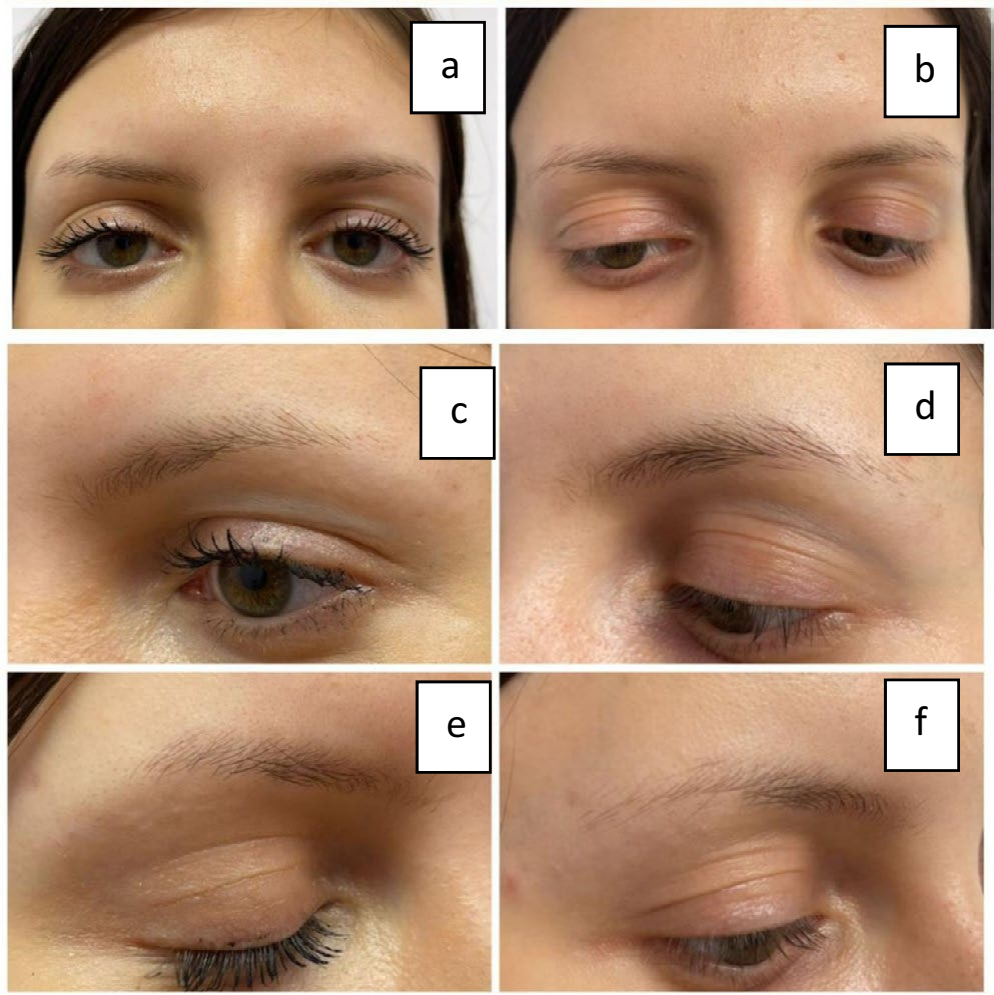
Clinical evaluation of a client with severe eyebrow loss response to EB MT. Figures a, c, and e represent the before first‐session–baseline from different perspectives: Front and side views; figures b, d, and f represent the after result from different perspectives: Front and side views.

Comparing the results of Figure 8, we can clearly see a positive improvement in EB after MT treatments. Three pictures on the left side represent the before state, whereas three pictures on the right represent the after results. Note that we are focusing on the inner part of the eyebrows (empty spaces in the eyebrows). The outer border of the eyebrows is not of interest since the consumers are subject to eyebrow shaping and thus the plucking of the hair follicles might have an overall impact. There is a strong positive outcome. Increased hair shaft diameter and increased EB density can clearly be assessed. It can be evaluated as an increase in both the volume and thickness of the eyebrows Figures 9, 10.

The goal of multicomponent serums is primarily to correct EB loss and it is designed to provide optimal conditions for cell biochemical processes by increasing the blood flow necessary to enhance the EB growth. The serums are rich in micronutrients, vitamins, minerals, and proteins that sufficiently supply the new hair follicles by directly opening the microchannels to the hair bulb—where newly developed hair follicles enter the anagen phase.

After the first MT session, we noticed a difference in the amount and strength of brow hair in clients. Some of the ingredients promote growth by creating a moisture‐capture barrier. But they can also increase circulation while stimulating cellular metabolism. This, in turn, promotes hair growth.

## 4 Conclusions

We have been performing hair MT for many clients thus far and have witnessed positive results including stimulation of resting hair follicles (which were in the telogen phase), stronger and thicker hair, intensely reduced hair fall, more hair volume, etc.

Based on photographic analysis and measurement, it was revealed that by the end of the EB MT protocol, despite the seen impact of the mesotherapy, there was a pronounced unseen impact of the application.

The unseen impact was the psychological boost that the consumer had after the sessions, such as more self‐confidence and more social interactions. Bearing in mind that physiologically speaking, the scalp hair and the EB hair are like each other, such tremendous positive effects hopefully will be seen in EB MT, and will prove to be an asset.

Our results suggest that there is a significant increase in EB growth due to direct hair growth stimulation of both the serums and the increased blood flow to the targeted area.

## Conflicts of Interest

The authors declare no conflicts of interest.

## Data Availability

The data that support the findings of this study are available on request from the corresponding author. The data are not publicly available due to privacy or ethical restrictions.

## Appendix A

Client Satisfaction Questionnaire.

Please, round the answer from 1 (very little) to 10 (extremely much).

1. How much are you satisfied with the results of eyebrow mesotherapy?
  1  2  3  4  5  6  7  8  9  10
2. How would you describe the pain level of eyebrow mesotherapy?
  1  2  3  4  5  6  7  8  9  10
3. Would you continue with the next sessions of eyebrow mesotherapy?
  YES  NO.
4. Would you recommend the eyebrow mesotherapy to the others?
  YES  NO.
5. How much do you feel that eyebrow mesotherapy has improved your overall appearance?
  1  2  3  4  5  6  7  8  9  10
6. Additional suggestions, criticisms or comments regarding the eyebrow mesotherapy.
  ________________________________________________.

Name & Surname __________________________.

Signature ___________________________.

Prishtina.

Date _____________.

## References

[1] B. Buffoli , F. Rinaldi , M. Labanca , et al., “The Human Hair: From Anatomy to Physiology,” International Journal of Dermatology 53 (2014): 331–341. 24372228 10.1111/ijd.12362

[2] V. Jennifer , “Nguyen MD the Biology, Structure, and Function of Eyebrow Hair,” Journal of Drugs in Dermatology 13, no. 1 (2014): s12–s16. 24385126

[3] E. B. G. De Koning , J. Passchier , and E. W. Dekker , “Psychological Problems With Hair Loss in General Practice and the Treatment Policies of General Practitioners,” Psychological Reports 67, no. 3 (1990): 775–778. 2287669 10.2466/pr0.1990.67.3.775

[4] C. Pratt Herbert , L. E. King , A. G. Messenger , et al., “Alopecia areata,” Nature Reviews. Disease Primers 3, no. 1 (2017): 1–37. 10.1038/nrdp.2017.11 PMC5573125 28300084

[5] M. Pistor , “What Is Mesotherapy?,” Le Chirurgien‐Dentiste de France 46 (1976): 59–60. 1076080

[6] M. Mammucari , A. Gatti , S. Maggiori , C. A. Bartoletti , and A. F. Sabato , “Mesotherapy, Definition, Rationale and Clinical Role: A Consensus Report From the Italian Society of Mesotherapy,” European Review for Medical and Pharmacological Sciences 15, no. 6 (2011): 682–694. 21796873

[7] G. Leibaschoff , “Mesotherapy and Cellulite,” American Journal of Mesotherapy 4 (2006): 53.

[8] A. Matarasso and T. M. Pfeifer , “Plastic Surgery Educational Foundation DATA Committee. Mesotherapy for Body Contouring,” Plastic and Reconstructive Surgery 115 (2005): 1420–1424. 15809611 10.1097/01.prs.0000162227.94032.ed

[9] H. Adelson , “French Mesotherapy for the Treatment of Pain,” American Journal of Mesotherapy 15 (2005): 21–23.

[10] L. Chen , D. Li , J. Zhong , et al., “Therapeutic Effectiveness and Safety of Mesotherapy in Patients With Osteoarthritis of the Knee,” Evidence‐Based Complementary and Alternative Medicine 2018 (2018): 6513049. 29507592 10.1155/2018/6513049 PMC5817326

[11] M. Majchrzycka , K. Adamska , K. Rachwalska , and Z. Adamski , “The Role of the Adjuvant Aesthetic Therapy in the Lives of Patients With Psoriatic Disease,” Advances in Dermatology and Allergology 39, no. 6 (2022): 1106–1109. 36686008 10.5114/ada.2022.118996 PMC9837584

[12] M. El‐Domyati , T. S. El‐Ammawi , O. Moawad , et al., “Efficacy of Mesotherapy in Facial Rejuvenation: A Histological and Immunohistochemical Evaluation,” International Journal of Dermatology 51, no. 8 (2012): 913–919. 22788806 10.1111/j.1365-4632.2011.05184.x PMC3513770

[13] T. Gorgulu , “An Alternative Treatment for Weakness and Sparseness of Eyebrows: Mesotherapy A Pilot Study,” Journal of Cosmetology & Trichology 1 (2015): 1000104.

[14] R. H. Weshahy , D. G. Aly , S. Shalaby , F. N. Mohammed , and K. S. Sayed , “Clinical and Histological Assessment of Combined Fractional CO_2_ Laser and Growth Factors Versus Fractional CO_2_ Laser Alone in the Treatment of Facial Mature Burn Scars: A Pilot Split‐Face Study,” Lasers in Surgery and Medicine 52, no. 10 (2020): 952–958. 32297661 10.1002/lsm.23252

[15] Y. Huang , F. Zhuo , and L. Li , “Enhancing Hair Growth in Male Androgenetic Alopecia by a Combination of Fractional CO_2_ Laser Therapy and Hair Growth Factors,” Lasers in Medical Science 32, no. 8 (2017): 1711–1718. 28528395 10.1007/s10103-017-2232-8

[16] A. Takeuchi , Y. Nomoto , M. Watanabe , S. Kimura , Y. Morimoto , and H. Ueda , “Application of Microneedles to Skin Induces Activation of Epidermal Langerhans Cells and Dermal Dendritic Cells in Mice,” Biological & Pharmaceutical Bulletin 39 (2016): 1309–1318. 27251665 10.1248/bpb.b16-00113

[17] A. Savoia , S. Landi , and A. Baldi , “A New Minimally Invasive Mesotherapy Technique for Facial Rejuvenation,” Dermatology and Therapy 3, no. 1 (2013): 83–93. 23888258 10.1007/s13555-012-0018-2 PMC3680640

